# Supplementary material for: Live-cell single-molecule tracking reveals co-recognition of H3K27me3 and DNA targets polycomb Cbx7-PRC1 to chromatin
Source: eLife. 2016 Oct 10;5:e17667. doi: 10.7554/eLife.17667 (PMC5056789; doi:10.7554/eLife.17667)
Supplement: Supplementary file 1. — DOI: http://dx.doi.org/10.7554/eLife.17667.064 [file elife-17667-supp1.docx]

**Supplementary file 1. Fractional sizes and diffusion constants of the CB, ID, and FD populations obtained from live-cell SMT analysis of the Cbx family proteins and their variants.**

|  | | CB population | | ID population | | FD population | | |
| --- | --- | --- | --- | --- | --- | --- | --- | --- |
|  |  | F_1_ | D_m1_ (µm^2^s^-1^) | F_2_ | D_m2_ (µm^2^s^-1^) | F_3_ | | D_m3_ (µm^2^s^-1^) |
| Wild-type mES cells | H2A-HaloTag | 72% ± 1% | 0.032 | 14% ± 2% | 0.50 ± 0.06 | 14% ± 2% | | 2.4 ± 0.2 |
|  | ^a^ HaloTag-NLS | NA | NA | 71% ± 1% | 0.24 ± 0.03 | 29% ± 2% | | 2.5 ± 0.1 |
|  | HaloTag-Cbx2 | 30% ± 1% | 0.032 | 61% ± 1% | 0.52 ± 0.05 | 9% ± 4% | | 1.6 ± 0.1 |
|  | HaloTag-Cbx4 | 10% ± 1% | 0.032 | 50% ± 1% | 0.32 ± 0.08 | 40% ± 2% | | 1.4 ± 0.1 |
|  | HaloTag-Cbx6 | 17% ± 1% | 0.032 | 47% ± 2% | 0.42 ± 0.08 | 36% ± 3% | | 1.5 ± 0.1 |
|  | HaloTag-Cbx7 | 29% ± 1% | 0.032 | 49% ± 1% | 0.32 ± 0.06 | 22% ± 3% | | 1.2 ± 0.2 |
|  | HaloTag-Cbx8 | 26% ± 1% | 0.032 | 50% ± 2% | 0.40 ± 0.06 | 24% ± 4% | | 1.2 ± 0.1 |
| *Cbx7^─/─^* mES cells | HaloTag-Cbx7 | 30% ± 1% | 0.032 | 46% ± 1% | 0.21 ± 0.02 | 24% ± 2% | | 1.1 ± 0.1 |
| *Eed^─/─^* mES cells | HaloTag-Cbx2 | 26% ± 1% | 0.032 | 54% ± 1% | 0.40 ± 0.08 | 20% ± 2% | | 1.6 ± 0.1 |
|  | HaloTag-Cbx4 | 14% ± 1% | 0.032 | 65% ± 1% | 0.38 ± 0.07 | 21% ± 2% | | 1.6 ± 0.1 |
|  | HaloTag-Cbx6 | 13% ± 1% | 0.032 | 52% ± 1% | 0.42 ± 0.09 | 35% ± 2% | | 1.5 ± 0.1 |
|  | ^a^ HaloTag-Cbx7 | NA | NA | 65% ± 3% | 0.26 ± 0.22 | 35% ± 6% | | 1.6 ± 0.6 |
|  | HaloTag-Cbx8 | 8% ± 2% | 0.032 | 40% ± 1% | 0.35 ± 0.17 | 52% ± 1% | | 1.3 ± 0.1 |
| *Ezh2^─/─^* mES cells | HaloTag-Cbx2 | 28% ± 1% | 0.032 | 41% ± 2% | 0.27 ± 0.14 | 31% ± 3% | | 0.9 ± 0.1 |
|  | HaloTag-Cbx4 | 13% ± 1% | 0.032 | 51% ± 2% | 0.37 ± 0.17 | 36% ± 2% | | 1.3 ± 0.1 |
|  | HaloTag-Cbx6 | 16% ± 1% | 0.032 | 44% ± 2% | 0.35 ± 0.17 | 40% ± 2% | | 1.4 ± 0.1 |
|  | HaloTag-Cbx7 | 12% ± 1% | 0.032 | 35% ± 3% | 0.35 ± 0.13 | 53% ± 2% | | 1.8 ± 0.1 |
|  | HaloTag-Cbx8 | 11% ± 1% | 0.032 | 40% ± 2% | 0.38 ± 0.10 | 49% ± 2% | | 1.4 ± 0.1 |
| *Y-Eed/Eed^─/─^* mES cells | HaloTag-Cbx7 | 29% ± 1% | 0.032 | 47% ± 2% | 0.35 ± 0.06 | 24% ± 2% | | 1.4 ± 0.1 |
|  | HaloTag-Cbx8 | 27% ± 1% | 0.032 | 40% ± 4% | 0.22 ± 0.20 | 33% ± 4% | | 1.1 ± 0.4 |
| *Y-Ezh2/Ezh2^─/─^* mES cells | HaloTag-Cbx7 | 30% ± 1% | 0.032 | 21% ± 1% | 0.14 ± 0.01 | 49% ± 1% | | 0.8 ± 0.1 |
|  | HaloTag-Cbx8 | 30% ± 1% | 0.032 | 45% ± 2% | 0.31 ± 0.01 | 25% ± 3% | | 1.1 ± 0.1 |
| *Ring1a^─/─^/Ring1b^─/─^* mES cells | HaloTag-Cbx7 | 44% ± 1% | 0.032 | 32% ± 2% | 0.25 ± 0.04 | 24% ± 2% | | 1.1 ± 0.3 |
| *Bmi1^─/─^/Mel18^─/─^* mES cells | HaloTag-Cbx7 | 40% ± 1% | 0.032 | 44% ± 1% | 0.28 ± 0.01 | 16% ± 5% | | 1.3 ± 0.1 |
| Wild-type mES cells | HaloTag-CD_Cbx7_ | 8% ± 1% | 0.032 | 54% ± 2% | 0.51 ± 0.17 | | 38% ± 2% | 1.8 ± 0.1 |
|  | HaloTag-Cbx7^F11A^ | 17% ± 1% | 0.032 | 42% ± 3% | 0.30 ± 0.14 | | 41% ± 4% | 1.2 ± 0.2 |
|  | HaloTag-Cbx7^ΔCD^ | 13% ± 1% | 0.032 | 44% ± 1% | 0.40 ± 0.17 | | 43% ± 2% | 1.6 ± 0.6 |
|  | HaloTag-Cbx7^ΔATL^ | 16% ± 1% | 0.032 | 44% ± 2% | 0.32 ± 0.06 | | 40% ± 3% | 1.5 ± 0.1 |
|  | HaloTag-Cbx7^ATLm^ | 16% ± 1% | 0.032 | 60% ± 1% | 0.43 ± 0.06 | | 24% ± 3% | 1.6 ± 0.1 |
|  | HaloTag-Cbx7^ΔCD-ATL^ | 8% ± 3% | 0.032 | 46% ± 1% | 0.35 ± 0.16 | | 46% ± 1% | 1.6 ± 0.1 |

^a^ Fit did not converge if the distributions were fitted with a three-component Gaussian function with the fixed D_m1_ = 0.032 µm^2^s^-1^.
