# Supplementary material for: Live-cell single-molecule tracking reveals co-recognition of H3K27me3 and DNA targets polycomb Cbx7-PRC1 to chromatin
Source: eLife. 2016 Oct 10;5:e17667. doi: 10.7554/eLife.17667 (PMC5056789; doi:10.7554/eLife.17667)
Supplement: Supplementary file 2. — DOI: http://dx.doi.org/10.7554/eLife.17667.065 [file elife-17667-supp2.docx]

**Supplementary file 2. Residence times, transient (F_1tb_) and stable (F_1sb_) chromatin-binding fractions of Cbx7 and its variants.**

| τ_lap_ = τ_int_ + τ_d_ τ_int_ = 0.03 s τ_d_ = 0.17 s | | | | | |
| --- | --- | --- | --- | --- | --- |
|  | | F_1tb_ population | | F_1sb_ population | |
|  |  | F_1tb_ | τ_tb_ (s) | F_1sb_ | τ_sb_ (s) |
| Wild-type mES cells | HaloTag-Cbx7 | 23.4% ± 1.1% | 0.79 ± 0.01 | 5.3% ± 0.4% | 7.3 ± 0.1 |
|  | HaloTag-CD_Cbx7_ | 6.6% ± 1.6% | 0.74 ± 0.02 | 1.5% ± 1.1% | 4.7 ± 0.1 |
|  | HaloTag-Cbx7^F11A^ | 13.6% ± 0.8% | 0.82 ± 0.01 | 3.0% ± 0.5% | 5.8 ± 0.1 |
|  | HaloTag-Cbx7^ΔCD^ | 10.8% ± 1.1% | 0.70 ± 0.01 | 2.1% ± 0.6% | 4.7 ± 0.1 |
|  | HaloTag-Cbx7^ΔATL^ | 13.2% ± 0.8% | 0.82 ± 0.01 | 2.9% ± 0.5% | 5.8 ± 0.1 |
|  | HaloTag-Cbx7^ATLm^ | 14.3% ± 1.3% | 0.73 ± 0.01 | 1.5% ± 0.5% | 6.0 ± 0.2 |
|  | HaloTag-Cbx7^ΔCD-ATL^ | 6.9% ± 1.4% | 0.71 ± 0.01 | 1.3% ± 0.9% | 4.6 ± 0.1 |
| *Ring1a^─/─^/Ring1b^─/─^* mES cells | HaloTag-Cbx7 | 34.7% ± 1.1% | 1.06 ± 0.02 | 9.6% ± 0.5% | 10.6 ± 0.2 |
| *Bmi1^─/─^/Mel18^─/─^* mES cells | HaloTag-Cbx7 | 32.2% ± 0.9% | 1.08 ± 0.02 | 7.8% ± 0.4% | 10.9 ± 0.1 |
