# Supplementary material for: Live-cell single-molecule tracking reveals co-recognition of H3K27me3 and DNA targets polycomb Cbx7-PRC1 to chromatin
Source: eLife. 2016 Oct 10;5:e17667. doi: 10.7554/eLife.17667 (PMC5056789; doi:10.7554/eLife.17667)
Supplement: Supplementary file 3. — DOI: http://dx.doi.org/10.7554/eLife.17667.066 [file elife-17667-supp3.docx]

**Supplementary file 3. U-track parameters used in this research.**

| **Step 1: Detection** | |  |
| --- | --- | --- |
| Gaussian standard deviation= 1.7 | |  |
| Camera bit depth = 16 | |  |
| Do Not Check "Use Rolling Window Time-Averaging" | |  |
| Do Not Check "Use Absolute Background" | |  |
| Check "Iterate to Estimate Gaussian Standard Deviation" | Maximum Number of Iterations: 10 |  |
| Check "Do Iterative Gaussian Mixture-Model Fitting" | Alpha Values: |  |
|  | Residuals = 0.05 | Distance = 0.05 |
|  | Amplitude = 0.05 |  |
| Frames to Use = 1 to 500 | |  |
| Do Not Check "View results immediately frame by frame" | |  |
| **Step 2: Tracking** | |  |
| Problem Dimensionality = 2 | |  |
| Maximum Gap to Close = 5 frames for population studies or 1 frame for residence time studies | |  |
| Minimum Length of Track Segments from First Step = 1 frames | |  |
| Check "Do segment merging" and "Do segment Splitting" | Note: Pop up window - Select Yes |  |
| Check "Plot histogram of gap lengths after gap closing" | |  |
| Check "Show calculation progress in command line" | |  |
| Do not check "Export tracking result to matrix format" | |  |
| Cost Functions Step 1 Setting | |  |
| Check "Allow directed motion position progration" | |  |
| Check "Allow instantaneous direction reversal" | |  |
| Brownian Search Radius | Lower Bound: 1 | Upper Bound: 10 |
| Multiplication Factor for Brownian Search Radius Calculation = 3 | |  |
| Check "Use nearest neighbor distance calculation" | |  |
| Number of frames = 10 | |  |
| Check "Plot histogram of linking distances | |  |
| Frame Numbers = 499 | |  |
| Cost Functions Step 2 and Kalman Filter Functions are unchanged | |  |
| **Step 3: Track Analysis** | |  |
| Motion Analysis Setting | |  |
| Problem Dimensionality = 2 | |  |
| Check "Check and analyze asymmetric flasks | |  |
| Alpha value for asymmetry determination = 0.1 | |  |
| Alpha value for moment scaling spectrum analysis = 0.05 | |  |
| Method for calculating the confinement radius: Mean positional standard deviation | | |
